# Effect of Health Literacy and Patient Activation on Health‐Seeking Behaviour: A Cross‐Sectional Study in Turkey

**DOI:** 10.1111/hex.70052

**Published:** 2024-10-07

**Authors:** Mehmet Akif Erişen

**Affiliations:** ^1^ Department of Healthcare Management, Erbaa Health Science Faculty Tokat Gaziosmanpaşa University Tokat Turkey

**Keywords:** health literacy, health‐seeking, patient, patient activation, Turkey

## Abstract

**Background:**

The study aimed to investigate the effect of health literacy and patient activation on health‐seeking behaviour. In addition, the role of general health status and age variables in this effect was also addressed.

**Methods:**

The research using quantitative methods is cross‐sectional. The participant information form, health literacy scale, patient activation scale and health‐seeking behaviour scale were used in the study. In addition to descriptive analyses, difference analysis, correlation analysis and multiple linear regression analysis were used in the study. The study participants consisted of 1045 adult individuals living in Turkey.

**Results:**

According to the correlation analysis results, it was determined that health literacy and patient activation were significantly positively associated with health‐seeking behaviour. According to multiple linear regression analysis, the independent variables of health literacy, patient activation, age and general health status significantly affected health‐seeking behaviour. Accordingly, patient activation and general health status positively affect health‐seeking behaviour, while health literacy and age have a negative effect.

**Conclusion:**

To positively improve individuals’ health‐seeking behaviours, it is thought that it would be appropriate to focus on individuals whose general health status is not good, who are not actively involved in their treatment, who have high health literacy and who are elderly.

**Patient or Public Contribution:**

Since the research examines the effect of patient activation and health literacy on health‐seeking behaviour, it is of great importance for the research that the public and patients are included in the study from the design of the research to the presentation of the findings. The emergence of the study was made possible by the public and patients’ evaluations of the research topics and their participation in the survey.

## 1 Introduction

The development of information technologies facilitates access to and use of information. This contributes to the formation of more literate societies in every field. In terms of health, developments in health services and technologies have improved the health structure of societies and increased lifespan. However, in addition to this, these developments may also bring some diseases. Patient participation and activation have an important place in the management of diseases. Therefore, advances in information technologies and health systems increase the importance of and interest in health literacy, health‐seeking behaviour and patient activation.

Health literacy is the capacity of people to read and understand health information and use it to make decisions. Decisions based on disinformation and misinformation can majorly impact people's health and safety [1]. In this sense, health literacy is important for people's health and safety. It is the extent to which individuals have the basic information they need to make decisions about health [2]. In addition, how they acquire, apply and understand this information is also related to health literacy [3]. It can also be defined as a set of skills that includes performing basic reading and numerical skills related to health services [4]. Health literacy is also closely related to individuals’ health‐seeking behaviours.

Health‐seeking behaviour aims to reveal the factors that enable or prevent individuals from making the right health‐related choices [5]. Health‐seeking behaviour can be narrowly defined as actions taken to restore well‐being when ill [6]. According to another source, health‐seeking behaviour refers to individuals seeking information about their health, such as risks, diseases and health‐protective behaviours to improve their health [7]. Individuals with Internet access can search for health information online, view personal records and communicate with health professionals. However, individuals with limited health literacy skills may need help navigating the website, understanding medical jargon and understanding which websites to trust based on the reliability of the health information presented [8]. In addition, another factor that will prevent them from experiencing these difficulties is the concept of patient activation.

Patient activation is the people's confidence, skills and knowledge in managing health and health services who actively participate in treatment [9]. Individuals can better manage their health through patient activation or patient engagement. An active patient can self‐manage problems, maintain a healthy state of being and engage in activities that reduce conditions that negatively affect their health, take part in treatment and diagnostic choices and cooperate in this process, choose the health institution by considering performance and quality, recognize the health system and thus have better health outcomes [10]. Patient activation is the ability and willingness of patients to take independent actions to manage their health [11]. A person who is more involved in the management of health is more informed, more aware of their health status, more confident in following medical prescriptions and more self‐effective [12, 13, 14]. Briefly, patient activation refers to being knowledgeable, skilled and confident in managing one's health care [12]. This is important because when patient activation is high, individuals confidently and proactively manage their health. They seek out information to make informed decisions and consistently engage in positive health‐related behaviours [15]. Patient activation is also associated with factors, such as individuals’ health literacy and quality of life. In their study with patients, Dunlay et al. [16] found that patients with low patient activation had low satisfaction levels and health literacy. Similarly, in the general population, higher activation is associated with healthier behaviours [17].

There are studies investigating the relationship between health literacy and health‐seeking behaviour [15, 18, 19, 20] or the relationship between patient activation and health‐seeking behaviour [21] on various samples. However, there is no study in which health literacy, patient activation and health‐seeking behaviour are addressed together. It is thought that health literacy may be related to health‐seeking behaviour, but patient activation also has an important share in this. Therefore, this study aims to examine the effect of health literacy and patient activation on health‐seeking behaviour. In other words, it was aimed to investigate whether individuals’ health literacy and their participation in their treatment (patient activation) affect health‐seeking behaviour. This study is thought to fill an important gap in the literature since it was conducted on a large sample living in Turkey and this issue has not been studied yet.

## 2 Materials and Methods

### 2.1 Design

In the study, quantitative methods were utilized. A survey and casual‐comparative research design were used in the study. This is a cross‐sectional study.

### 2.2 Population and Sample

The population of the study consists of adults living in Turkey. According to TurkStat [22], as of the end of 2022, approximately 63 million of Turkey's population of 85 million people consisted of adults. According to Gürbüz and Şahin [23], for population sizes of more than one million, a sample of at least 384 people with 95% reliability and a sample of at least 665 people with 99% reliability is sufficient to represent the population. In the research, 1045 adult individuals were reached by using the convenience sampling method. Since the study was conducted through online social media groups, individuals who use social media, are over the age of 18 and live in Turkey were included in the study. The questionnaire form was sent to social media groups by the researchers and the people in these groups who were willing to participate in the study completed the questionnaire form. The fact that social media is mostly used by young people explains why approximately 62% of the research sample consists of individuals under the age of 30. It can be said that the fact that the research was conducted online through social media groups constitutes a limitation in terms of reaching older individuals less and concentrating the sample on the younger group.

### 2.3 Data Collection Tools

The research data were collected online through Google Forms between March and June 2023. The link to the survey form was delivered to adult individuals living in Turkey using a convenience sampling method through social media groups. Participation in the survey was completely voluntary and the participants’ consent to participate was obtained through the consent form attached at the beginning of the survey before filling out the survey form.

The questionnaire form consists of four parts: participant information form, health literacy scale, patient activation scale and health‐seeking behaviour scale.

#### 2.3.1 Participant Information Form

The participant information form in the first part consists of eight questions, including age, gender, education level, marital status, chronic disease status of self and family members, medication use and general health status. General health status was measured by a Visual Analogue Scale (VAS) with a range of 1 (very poor) to 10 (very good) asking patients how they perceived their own health status. However, this is not a validated item, so it can have some limitations.

#### 2.3.2 Health Literacy Scale

The health literacy scale was developed in English by Sorensen et al. [24] to determine the health literacy level of individuals. The language, content and construct validity of the Turkish adaptation of the scale was conducted by Aras and Bayık Temel [25]. The health literacy scale is a five‐point (between 1 and 5) Likert‐type scale and consists of 25 items. A minimum score of 25 and a maximum score of 125 can be obtained from the scale. The scale measures individuals’ level of access, understanding, evaluation and utilization of information about their health. As the score obtained from the scale increases, health literacy increases, and as it decreases, health literacy decreases. The internal consistency coefficient of the scale was found to be 0.92 in the adaptation study and 0.99 in the current study.

#### 2.3.3 Health‐Seeking Behaviour Scale

The health‐seeking behaviour scale was developed by Kıraç and Öztürk [26] to determine the health‐seeking behaviour of individuals. The health‐seeking behaviour scale is a five‐point (between 1 and 5) Likert‐type scale and consists of 12 items. A minimum score of 1 and a maximum score of 5 can be obtained from the scale. The scale measures individuals’ tendency to seek information about their illness from various sources. As the score obtained from the scale increases, health‐seeking behaviour increases, and as the score decreases, health‐seeking behaviour decreases. The internal consistency coefficient of the scale was found to be 0.75 in the original study and 0.96 in the current study.

#### 2.3.4 Patient Activation Scale

Patient activation scale was developed by Hibbard et al. [10], adapted into a short form by Hibbard et al. [27], and adapted into Turkish by Koşar [28] to determine and evaluate the level of patient activation. The patient activation scale is a five‐point (between 0 and 4) Likert‐type scale and consists of 13 items. A minimum score of 0 and a maximum score of 52 can be obtained from the scale. The scale measures individuals’ level of involvement in and control over their own health processes. The higher the score on the scale, the higher the patient activation and the lower the score, the lower the patient activation. The internal consistency coefficient of the scale was found to be 0.81 in the adaptation study and 0.97 in the current study.

### 2.4 Analysis of the Data

The analysis of the research data was carried out using the IBM SPSS 26 package programme. After normality tests of the scales, it was seen that the skewness coefficients were between −0.06 and −1.61, and the kurtosis values were between −1.04 and 1.90. Plitcha and Kelvin [29] stated that there is a normal distribution when the skewness value is in the range of ±1.96. In this context, it was assumed that the data were normally distributed in this study. Cronbach's *α* test, descriptive statistical analyses, independent sample *t* test, one‐way analysis of variance (ANOVA), Pearson correlation analysis and multiple linear regression analysis were performed in the SPSS 26 package programme. According to Gürbüz and Şahin [23], the correlation coefficient in Pearson correlation analysis shows a low relationship between 0 and 0.3, a moderate relationship between 0.3 and 0.7 and a strong relationship between 0.7 and 1. Gender, marital status, educational status, chronic disease status and continuous medication use were not included as independent variables in the multiple linear regression model. There is no basis in the literature that gender, marital status and educational status affect health‐seeking behaviour. Therefore, these independent variables were not included in the model. On the other hand, VIF values are high when chronic diseases and continuous medication use are included in the model since they act in a similar way to the age variable. Therefore, only age and general health status were included in the model among the descriptive variables. As a result of the Pearson correlation analysis, the level of relationship between the variables is below 0.70 and the VIF values and Durbin–Watson coefficient are within normal values in the regression model, indicating that it is appropriate to perform multiple linear regression analysis on this model. In addition, the evaluation of the research findings was carried out at a 95% confidence interval and *p* < 0.05 significance level.

### 2.5 Ethics Approval

The ethics committee's permission for the research was obtained from Tokat Gaziosmanpaşa University Social and Human Sciences Research Ethics Committee on 04.04.2023 with decision numbers 01–17. In addition, consent for participation in the survey was obtained from the research participants before filling out the questionnaire form. The research was conducted in accordance with the Declaration of Helsinki.

## 3 Results

This section presents descriptive findings of the participants, descriptive results of the scales, Pearson correlation analysis results and multiple linear regression analysis results.

The descriptive information about the participants is shown in Table 1. Accordingly, 61.5% of the participants were female, 46.6% were between the ages of 21–30, 53.5% were unmarried and 50.2% had a high school education. In addition, 69.8% of the participants did not have any chronic disease, while 29.2% stated that they were constantly taking medication. On the other hand, 45.4% of the participants reported living with a family member diagnosed with a chronic disease.

**Table 1:** Descriptive statistics of the participants.

Descriptive variables	*n*	%
Gender		
Female	643	61.5
Male	402	38.5
Age		
18–20	162	15.5
21–30	487	46.6
31–40	191	18.3
41–50	85	8.1
51–64	56	5.4
65 +	64	6.1
Marital status		
Married	486	46.5
Not married	559	53.5
Education level		
Primary education and below	168	16.1
High school	525	50.2
Associate's degree	121	11.6
Bachelor's degree	184	17.6
Postgraduate	47	4.5
Chronic disease		
No chronic disease	729	69.8
Related to the respiratory system	13	1.2
Related to the cardiovascular system	117	11.2
Related to the endocrine system	155	14.8
Related to the muscle, bone or nervous system	31	3.0
Medication used continuously		
Yes	305	29.2
No	740	70.8
Presence of chronic disease in the family (excluding self)		
Yes	474	45.4
No	571	54.6

*Note: n* = 1045.

In Table 2, the scores obtained from health literacy, health‐seeking behaviour and patient activation scales were compared according to some characteristics of the participants. Independent sample *t* test analysis was used to compare the scale scores. Accordingly, it was found that there was no significant difference in health literacy, health‐seeking behaviour and patient activation scores according to the gender of the participants (*p* > 0.05). While no significant difference was found in the health literacy and patient activation scores of the participants according to their marital status (*p* > 0.05), it was observed that unmarried participants had significantly higher scores in health‐seeking behaviours than married participants (*p* < 0.05). When examined in terms of chronic disease and continuous medication use, it was found that the health literacy, health‐seeking behaviour and patient activation scores of participants who did not have a chronic disease and did not use continuous medication were significantly higher (*p* < 0.05). Finally, it was found that there was a significant difference in health literacy, health‐seeking behaviour and patient activation scores according to the age of the participants (*p* < 0.05). It is seen that the difference in health literacy and health‐seeking behaviour scores is generally because younger participants have higher scores than older participants. In addition, the difference in patient activation is due to the fact that individuals aged 21–30 years have higher scores than aged 18–20 years. In addition, although not significant, it was also noteworthy that the patient activation score of the participants between the ages of 21–30 was higher than the older groups.

**Table 2:** Comparison of scale scores according to participant characteristics.

Independent variable	Health literacy		Health‐seeking behaviour		Patient activation	
	Mean	SD	Mean	SD	Mean	SD
Gender						
Female	97.10	28.64	3.24	1.14	40.86	10.92
Male	100.48	27.80	3.16	1.26	41.74	11.37
t		−1.891		0.961		−1.246
*p*		0.059		0.337		0.213
Marital status						
Married	96.90	29.40	2.89	1.03	41.71	8.61
Not married	99.70	27.37	3.48	1.25	40.76	12.88
t		−1.586		−8.330		1.409
*p*		0.113		< 0.001***		0.159
Presence of chronic disease						
Yes	85.54	37.71	2.65	1.00	39.74	10.56
No	103.97	20.85	3.45	1.18	41.84	11.28
t		−8.162		−11.186		−2.892
*p*		< 0.001***		< 0.001***		0.004**
Continuous medication use						
Yes	84.79	38.76	2.55	1.00	39.75	10.94
No	104.01	20.24	3.48	1.15	41.80	11.12
*t*		−8.210		−13.018		−2.717
*p*		< 0.001***		< 0.001***		0.007**
Age						
18–20 ^1^	98.12	26.90	3.51	1.37	37.88	15.08
21–30 ^2^	104.70	19.02	3.54	1.09	42.59	9.84
31–40 ^3^	102.19	22.06	3.03	1.12	40.75	11.40
41–50 ^4^	92.87	34.26	2.63	0.97	40.64	9.92
51–64 ^5^	70.38	44.25	2.18	0.68	40.66	9.83
65 + ^6^	71.75	46.13	2.14	0.57	41.66	7.92
*F*		32.696		39.581		4.658
*p*		< 0.001***		< 0.001***		< 0.001***
Post hoc		1,3,4 > 5,6; 2 > 1,4,5,6		1,2 > 3,4,5,6; 3 > 4,5,6; 4 > 5,6		1 < 2

Abbreviation: SD, standard deviation.

** *p* < 0.01.

*** *p* < 0.001.

Mean, SD and Pearson correlation results of variables are shown in Table 3. The mean values were 102.63 ± 29.54 for health literacy, 3.21 ± 1.19 for health‐seeking behaviour, 41.20 ± 11.10 for patient activation, 31.98 ± 13.44 for age and 7.40 ± 2.19 for general health status. There was a low level and positive and significant relationship (*r* = 0.193) between health literacy and health‐seeking behaviour of the participants, and there was a medium level positive and significant relationship (*r* = 0.453) between health literacy and patient activation. In addition, a significant positive relationship (*r* = 0.357) was found between health‐seeking behaviour and patient activation at a moderate level. There was a significant medium‐level negative relationship between age and health literacy (−0.317), health‐seeking behaviour (*r* = −0.389) and general health status (*r* = −0.450). In addition, a significant positive relationship was found between general health status and health literacy (*r* = 0.241), health‐seeking behaviour (*r* = 0.248) and patient activation (*r* = 0.080) at a low level.

**Table 3:** Mean, SD and Pearson correlation results of variables.

Variables	Mean	SD	1	2	3	4
1. Health literacy	102.63	29.54				
2. Health‐seeking behaviour	3.21	1.19	0.193**			
3. Patient activation	41.20	11.10	0.453**	0.357**		
4. Age	31.98	13.44	−0.317**	−0.389**	0.016	
5. General health status	7.40	2.19	0.241**	0.080*	0.248**	−0.450**

*Note: n* = 1045.

* *p* < 0.05.

** *p* < 0.01.

**Figure 1:**
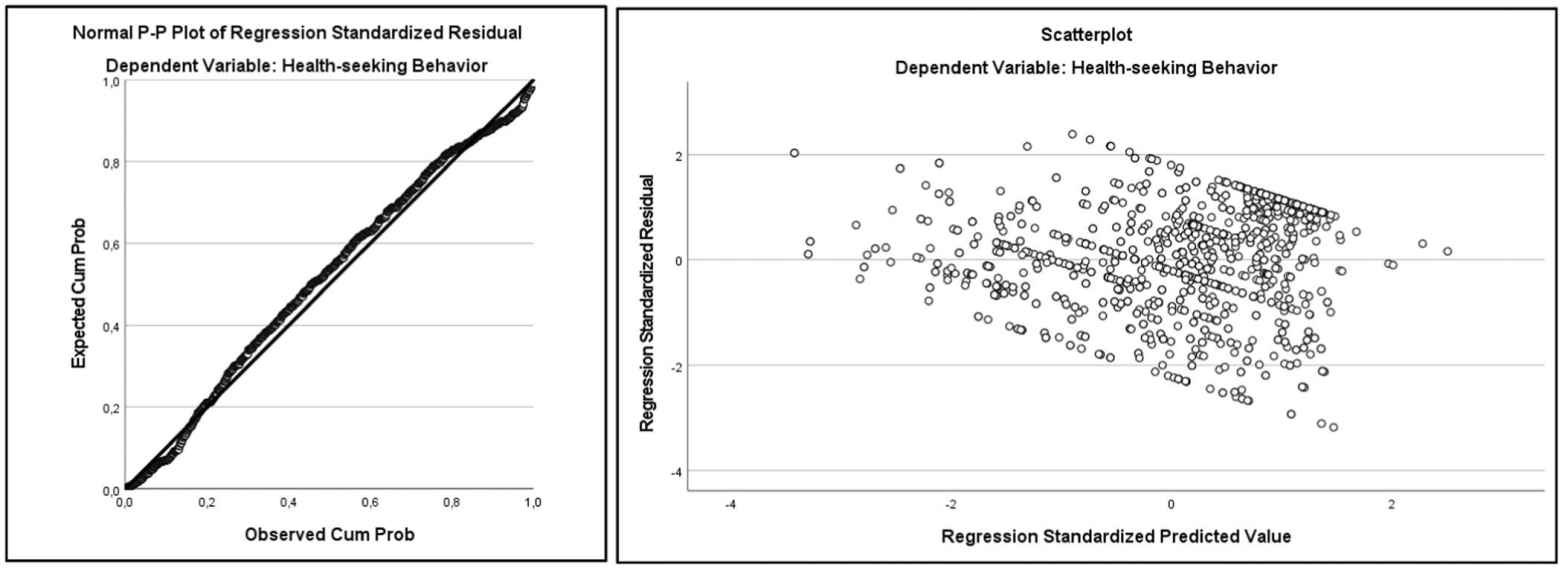
Graphs for multiple linear regression assumptions.

Figure 1 shows the graphs for multiple linear regression assumptions. The differences between the observed and predicted values of the dependent variable are examined in the Predicted Probability (P‐P) plot. It was observed that the normal distribution of the residuals was closely aligned with the diagonal line of the graph, thus normality was ensured. Homoscedasticity is examined through the scatter plot of the estimated values against the residuals. Accordingly, there are equally distributed points above and below zero on the X‐axis and to the left and right of zero on the Y‐axis. Moreover, according to Table 4, the Durbin–Watson coefficient of the model is calculated as 1.174, indicating that there is no autocorrelation. On the other hand, the variance inflation factor (VIF) values of the independent variables are between 1.27 and 1.45. This shows that there is no multicollinearity among the variables. When all these situations are taken together, it can be said that the assumptions of multiple linear regression are met for the research model.

**Table 4:** Multiple linear regression analysis results.

Independent variables	*B*	S E	*β*	95% CI for *B*		*t*	*p*	VIF	*R*	*R* ^2^_Adj._	*F*	*p*
				Lower	Upper							
(Constant)	2.864	0.215		2.441	3.286	13.288	< 0.001***		0.549	0.298	112.056	< 0.001***
PA	0.046	0.003	0.426	0.039	0.052	14.395	< 0.001***	1.30				
HL	−0.006	0.001	−0.150	−0.009	−0.004	−4.778	< 0.001***	1.45				
GHS	0.034	0.016	0.063	0.003	0.065	2.160	0.031*	1.27				
Age	−0.037	0.003	−0.415	−0.042	−0.031	−13.628	< 0.001***	1.38				

*Note: R* ^2^ = 0.301, Durbin–Watson = 1.174, Dependent Variable = Health‐Seeking Behaviour.

Abbreviations: HL, health literacy; GHS, general health status; PA, patient activation.

* *p* < 0.05.

*** *p* < 0.001.

Table 4 shows the multiple linear regression analysis conducted to predict the health‐seeking behaviour variable using the independent variables of patient activation, health literacy, general health status and age. According to the results of the analysis, a significant regression model (*F* = 112.056, *p* < 0.001) was found in which the independent variables included in the model explained 29.8% of the health‐seeking behaviour. Among the independent variables in the model, patient activation positively and significantly explains health‐seeking behaviour (*β* = 0.426, *t* = 14.395, *p* < 0.001). Health literacy explains health‐seeking behaviour negatively and significantly (*β* = −0.150, *t* = −4.778, *p* < 0.001). General health status explains health‐seeking behaviour positively and significantly (*β* = 0.063, *t* = 2.160, *p* = 0.031). Finally, the age of the participants explains health‐seeking behaviour negatively and significantly (*β* = −0.415, *t* = −13.628, *p* < 0.001).

## 4 Discussion

Day by day, the development of health systems and new treatment methods, in addition to the prolongation of life expectancy in societies and the increase in chronic diseases, make it necessary for society to raise awareness and participate in providing health services. Therefore, individuals’ health literacy, patient activation and health‐seeking behaviours become important issues. In this context, the study aims to examine the impact of patient activation and health literacy on health‐seeking behaviour.

In the study, it was found that the health literacy of the participants did not differ significantly according to gender and marital status, but a significant difference was found in the variables of chronic disease, continuous medication use and age. It was observed that the health literacy of people who did not use continuous medication and did not have chronic diseases was higher. In addition, it was found that participants whose age was relatively younger had higher health literacy. Colbert et al. [30] found that health literacy did not differ significantly according to gender and marital status. Similarly, Garcia‐Codina et al. [31] found no significant difference in health literacy scores according to gender, while participants who were younger and did not have chronic diseases had higher health literacy. Ozdemir et al. [32] stated that younger participants had higher health literacy. In this study, the health‐seeking behaviours of the participants did not differ significantly according to gender, but those who were not married, those who did not have chronic diseases, those who did not have to take medication continuously and those who were relatively young had higher health‐seeking behaviours. According to Şantaş et al. [33], there was no significant difference in the health‐seeking behaviours of the participants according to gender and marital status. However, although there was no significant difference, it was also noted that unmarried participants had higher health‐seeking behaviours. In addition, participants younger than 40 years of age, similar to this study, and people with chronic diseases, unlike this study, had higher health‐seeking behaviours. Kıraç [34] stated in his study that people who do not have chronic diseases and do not use medication continuously have higher health‐seeking behaviour. Çankaya and Filiz [35] revealed that people without chronic diseases have significantly higher health‐seeking behaviour, and those who do not use continuous medication have higher health‐seeking behaviour, although not at a significant level. Finally, it was revealed that the patient activation of the participants did not differ significantly according to gender and marital status variables. In addition, the patient activation scores of those who do not have chronic diseases and those who do not use continuous medication were found to be significantly higher. It was also revealed in the study that the patient activation of participants between the ages of 21–30 was higher. According to Yayla [36] and Çiftçi Kıraç and Ertaş [37], there was no significant difference in patient activation scores according to gender and there was a significant difference/relationship in the age variable. However, in the analyses conducted according to the variables of marital status, chronic disease and continuous medication use, results contrary to this study were revealed. In addition to all these, in this study, it is thought that the score differences in the scales in favour of those who do not have a chronic disease and do not use continuous medication are mostly due to age status. It has been observed that the participants who constantly use medication and have chronic diseases are mostly composed of individuals of higher age.

In the study, a low‐level positive relationship was found between health literacy and health‐seeking behaviour. Studies investigating the relationship between health‐seeking behaviour and health literacy have also found that these two variables are positively correlated with each other [33, 38]. In addition, Bak et al. [39] stated that individuals with adequate health literacy can distinguish misinformation in health‐seeking behaviour more easily, so they are better equipped for health‐seeking behaviour. Individuals with inadequate health literacy may continue to be misled by inaccurate sources of health information and may have difficulty navigating health‐seeking behaviours [40]. Therefore, it can be stated that health literacy is very important if patients are to benefit from health services [38]. Nielsen‐Bohlman et al. [3] stated that the health literacy level of individuals can affect their health status by affecting their health service‐seeking behaviours. In addition, the study showed that patient activation was positively and moderately associated with health literacy and health‐seeking behaviours. Studies in the literature reveal a positive relationship between health knowledge level or health literacy and patient activation [36, 41]. Another study found that self‐efficacy, which is related to health literacy [42], was positively associated with patient activation [12]. These findings support the findings of this study.

Although it has been previously determined that health literacy and patient activation affect health‐related quality of life [36], it is thought that examining the effect of these two concepts on health‐seeking behaviour will fill an important gap in the literature. According to the multiple linear regression analysis conducted in this direction, the effect of patient activation and health literacy on health‐seeking behaviour was found to be statistically significant. While the increase in patient activation increased health‐seeking behaviour, the increase in health literacy had a negative effect on health‐seeking behaviour. In addition, general health status and age variables included in the model were also found to have a significant effect on health‐seeking behaviour. It was determined that health‐seeking behaviour was positively affected as general health status improved, and health‐seeking behaviour was negatively affected as age increased. The measurement of general health status in the study can be considered as a limitation since it was carried out with a measurement that has not been validated. But Espinosa and Espinosa [43] found that preventive and curative health‐seeking behaviours are positively associated with quality of life, which is related to general health perception [44]. This finding can be considered as a supportive situation for the research result. On the other hand, activated patients are more likely to adhere to treatment regimens and have better clinical outcomes [45]. Indeed, Graffigna et al. [21] view health‐seeking behaviour as a goal‐oriented and purposeful activity through patient engagement. Therefore, they find that patient activation is an essential factor influencing an individual's health information‐seeking behaviour. People with high activation levels are individuals who have the necessary knowledge and skills to take responsibility for their health to maintain their health and improve their well‐being [37]. Weaver et al. [46] stated a positive relationship exists between information‐seeking behaviour on healthy living and self‐reported health awareness or being active in health. Gutierrez et al. [8] reported that health literacy has no significant effect on health information‐seeking behaviour. On the other hand, Gorczynski et al. [47] found that people with high health literacy have higher health‐seeking behaviour. Lee et al. [48] emphasized that this relationship between health literacy and health‐seeking behaviour depends on access to technological devices. The striking point in this study is that there is a significant positive relationship between health literacy and health‐seeking behaviour in correlation analysis. However, when evaluated together with patient activation, age and general health status variables in the multiple linear regression model, the effect of health literacy on health‐seeking behaviour turns negative. This indicates that the health literacy of patients will not continue to positively affect health‐seeking behaviour when evaluated together with patient activation, age and general health status. It is thought that the negative effect here may be because people who are young, active in their health and have a high general health status are more self‐confident about their health and do not feel the need to seek health when their health literacy is also high. In other words, when individuals who are young, active and perceive their general health as good have high health literacy; it is thought that there may be a decrease in health‐seeking behaviour with the idea that ‘I am young, I can manage my health and I can overcome my illness with my own knowledge and experience’. Of course, it will be possible to clearly understand the reason for this by conducting qualitative interviews as well as quantitative interviews with the participants using a mixed model.

## 5 Conclusion

Health literacy and patient activation have a positive relationship with health‐seeking behaviour when considered separately correlationally. But in a situation where four variables are considered together, high health literacy should not be considered as an issue that will increase health‐seeking behaviour, the role of patient activation, age and general health status should also be taken into consideration here. Therefore, policy makers should encourage practices and technologies that will increase patient activation and engagement in addition to policies aimed at increasing individuals’ health literacy. In other words, increasing the health literacy of individuals will certainly be beneficial in terms of social awareness; at the same time, patients’ information‐seeking behaviours related to their health can be positively improved by ensuring that they are included in their own health processes, more actively in this regard. This could be an important step in creating healthier societies. In doing so, they should also take into account their age and general health status.

## Author Contributions

**Mehmet Akif Erişen:** Conceptualization, methodology, formal analysis, data curation, investigation, visualization, writing–original draft, writing–review & editing.

## Ethics Statement

The ethics committee's permission for the research was obtained from Tokat Gaziosmanpaşa University Social and Human Sciences Research Ethics Committee on 04.04.2023 with decision numbers 01‐17.

## Consent

Consent for participation in the survey was obtained from the research participants before filling out the questionnaire form.

## Conflicts of Interest

The authors declare no conflicts of interest.

## Data Availability

The data underlying this article will be shared on reasonable request to the corresponding author.
